# The effect of viscosity and surface tension on inkjet printed picoliter dots†

**DOI:** 10.1039/c9ra04993b

**Published:** 2019-10-07

**Authors:** Sarah Krainer, Chris Smit, Ulrich Hirn

**Affiliations:** Institute of Paper, Pulp and Fiber Technology, TU Graz Inffeldgasse 23 8010 Graz Austria ulrich.hirn@tugraz.at +43 316 87330753; CD Laboratory for Fiber Swelling and Paper Performance Inffeldgasse 23 8010 Graz Austria; Océ Van der Grintenstraat 1 5914 HH Venlo Netherlands

## Abstract

In this study, we investigated the effect of liquid viscosity and surface tension for inkjet printing on porous cellulose sheets. We used five model liquids, representing the operational field of an industrial high speed inkjet printer, as specified by Ohnesorge- and Reynolds number. Drops with 30 pl and 120 pl drop size were jetted with a commercial HSI printhead. We printed on four uncoated papers representing the most relevant grades on the market in terms of hydrophobisation and surface treatment. We are presenting a quantitative analysis of viscosity and surface tension on the print outcome, evaluating dot size, liquid penetration (print through) and surface coverage of the printed dots. The most important finding is that for liquids within the jetting window the variation of the liquid viscosity typically has a 2–3 times higher impact on the print outcome than variation of the liquid surface tension. Increased viscosity in all cases reduces dot area, liquid penetration and liquid surface coverage. Surface tension plays a smaller role for liquid spreading and penetration, except for hydrophobised substrates, where both are reduced for higher surface tension. Interestingly, higher surface tension consistently increases liquid surface coverage for all papers and drop sizes. A detailed analysis on the competing effect of dot spreading and liquid penetration is presented, in terms of viscosity, surface tension and surface coverage of the liquid.

## Abstract 2

In this study, we investigated the effect of liquid viscosity and surface tension for inkjet printing on porous cellulose sheets.

## Graphical abstract

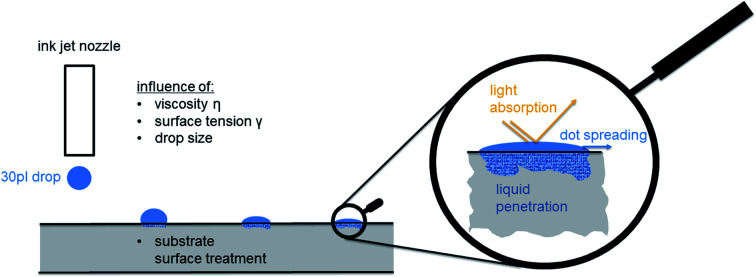

## 1 Introduction

Inkjet printing on porous substrates covers a wide field of applications, from printing on paper^1,2^ and other flexible substrates^3–5^ to fields like metal patterning,^6–8^ film creation for solar cells,^9,10^ printing polymer solutions droplets^11,12^ or hydrophobisation of surfaces.^13^ When printing on porous substrates the resulting printing dot is affected by two competing processes, the spreading of the drop on the substrate, and the penetration of the drop into the substrate, hence both are relevant for inkjet printing.^14,15^

Capillary penetration of liquids is often taken as a model for liquid imbibition into porous sheets. The Lucas Washburn equation^16^ and the Bosanquet equation^17^ are commonly employed, where the latter is a generalization of the former. While the Lucas Washburn equation only considers capillary and viscous forces, the Bosanquet equation also takes inertial forces into account, a detailed analysis provides *e.g.* Schoelkopf.^18^ However, the absolute experimental data reveal huge quantitative and also qualitative deviations from the calculated penetration.^19^ Hence, there is ongoing refinement of these models, *e.g.* by introduction of modification factors,^20–22^ like the dynamic contact angle.^23–25^ Anyway, the pore system in sheet material like paper is much more complex in terms of geometry and interconnectivity than the circular tube geometry assumed for the above mentioned model equations. This is the reason why numerical simulation of pore flow is a highly relevant and increasingly important approach here.^26–29^

Analysis of drop spreading and penetration for inkjet printing is challenging due to several factors. First, the relevant drop size is in the low picoliter range, with a drop diameter around 24 μm. Yet it is common to use microliter drops^30,31^ for analysis of contact angle and liquid penetration, a size scale larger by the factor 10^6^. Also evaporation plays a big role, due to the small inkjet drop size, it is limiting capillary penetration.^32^ In high speed inkjet printing, a technology that is increasingly important for industrial printing, the ink setting on a porous media also happens very fast, usually within 30–100 milliseconds.^33^ Capturing the relevant time- and size scale in the experimental investigations is very relevant, evaluating too large drops results in a gross oversupply on liquid on the sheet surface and in the sheet bulk, which results in unrealistic drop spreading and much too slow penetration.

Several investigations were addressing the influence of viscosity as well as surface tension on ink penetration and wetting, without capturing it quantitatively.^34,35^ Here, viscosity and surface tension play a role in evaporation and penetration. An effective, increased viscosity of a water/glycerol mixture leads to a deviating penetration behaviour^34^ and slows down the liquid flow.^35^ Han and coworkers^36^ found, that the higher the ratio of surface tension to viscosity is, the higher is the filling rate. The viscosity also plays a major role in the inertial selection of pores at the beginning of penetration^37,38^ and during the surface wetting process.^18,39–41^ Furthermore, Neogi *et al.*^42^ showed that also the spreading rate of a liquid drop depends on the surface tension and viscosity.

Not only the liquid properties are responsible for wetting and penetration, but also the substrate. A lot of experimental research was done evaluating the influence of pore-size and the surface properties of liquid behaviour on cellulose material. The porous media bulk property (porosity and average pore size) plays a major role in penetration,^18,19,40,41,43^ the bigger the initial pore diameter the stronger is the influence of inertia. Also the surface chemistry of the substrate has a large influence. Matching polarity of the liquid and the substrate as well as a high surface energy of the sheet material lead to stronger wetting, *i.e.* it promotes drop spreading and liquid penetration. Both of that is indicated by a lower contact angle between drop and sheet.^44^

Penetration of the color into the paper bulk reduces color density and increases print through. Hence in surface treatment for inkjet printing a common goal is to retain the coloring material on the surface, while it is necessary that ink vehicle penetrates to the paper bulk to immobilize the ink layer. This may be achieved by treating a paper surface with bivalent salts which breaks down the dispersion of pigment inks and leads to precipitation of the pigments on the surface.^45^ Another approach for surface treatment is application of cationic material. This has been shown to decrease the ink penetration, but also reduces bleeding of an ink droplet.^1,46,47^

## 2 Aim of the work

As outlined in the introduction there are several important factors determining drop spreading and drop penetration in inkjet printing. Those are viscosity and surface tension of the liquid, pore system of the substrate, and the wetting behavior (contact angle) of the liquid on the substrate, which is defined by the interplay of cohesion forces in the liquid and its adhesion to the surface. The literature in the field has been addressing some of these influence factors. However, in order to draw valid conclusions for the inkjet printing process it is necessary that the research is

• considering the correct size scale, *i.e.* picoliter drops like in actual inkjet printing

• considering the correct time scale, *i.e.* setting of the liquid within the first second after drop impact

• considering liquids within the actual window of operation for inkjet printing

• considering relevant substrates.

While most of the work in the literature is considering one or maybe two of these requirements, a systematic study of the driving factors on inkjet print outcome under real process conditions is missing. This paper is aiming to fill some of that gap. It addresses the influence of liquid viscosity and surface tension on the print outcome for the industrial high speed inkjet (HSI) printing process. We are evaluating drop spreading, liquid penetration and light absorption of dots, the printing has been carried out on a test setup employing commercial high speed inkjet (HSI) printheads. We use drops in the relevant size scale, which is in the low picoliter range (30 pl and 120 pl), having liquid setting in the HSI printing time scale. This is a specific advantage of our approach in comparison to other state of the art measurement techniques. The printed liquids have been developed in such a way that viscosity and surface tension is varied as much as possible within the operating window of the printhead. As test substrates, we are using papers representing the most relevant paper grades in the market for uncoated inkjet printing papers. In this way we are able to quantify the influence of liquid surface tension and viscosity on the printing result for realistic inkjet conditions.

## 3 Materials and methods

### 3.1 HSI printhead and drop volume

For our print tests we employed a commercial inkjet print-head, like it is used in the Océ Varioprint i300 printer. It is a piezoelectric printhead, which shoots the drops on demand (Drop-on-demand method) and has a resolution of 600 dpi.^48^ The model liquid drop size can be set up to a volume of 29 pl. The printhead employs frequencies of up to 30 kHz. In this work all printing tests have been carried out with 30 pl drops and 120 pl drops. We generate droplet volumes of 120 pl by shooting 4–6 droplets in a row, which coalesce to one drop before they impinge on the paper surface. For detailed information on drop volume please have a look in the ESI.†

#### 3.1.1 Operational field of the HSI printhead

**Fig. 1:**
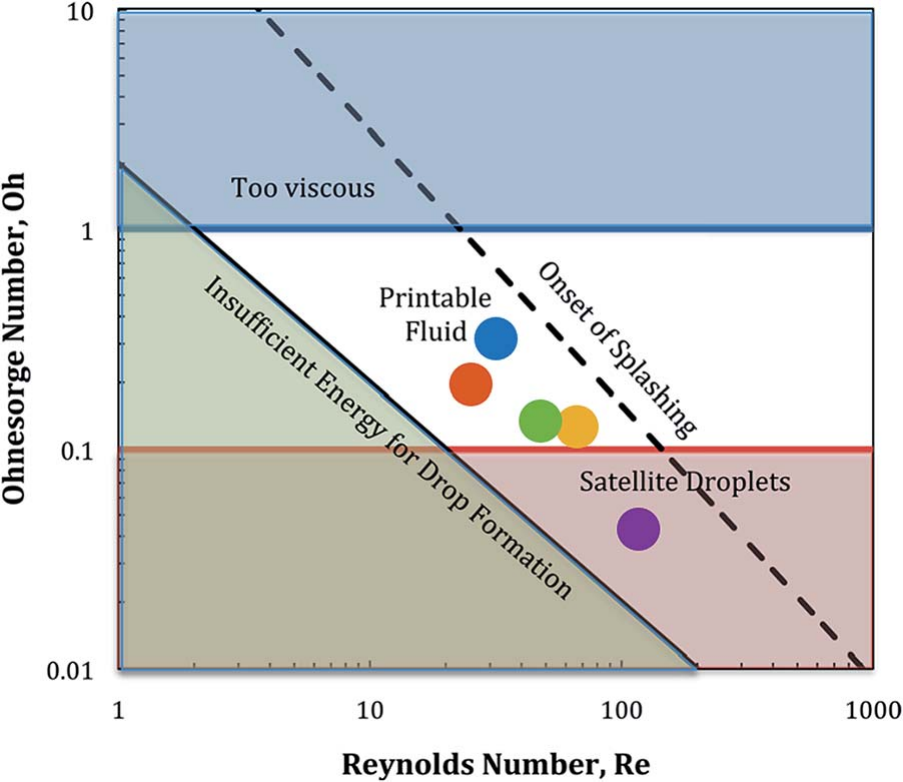
Ohnesorge number plotted over Reynolds number, indicating the printable fluid window.^49^ The dots indicate the position of the testing liquids used in this work.

The Ohnesorge number (Oh), eqn (1), and the Reynolds number (Re), eqn (2), are dimensionless parameters, which can be used to indicate the jettability of a liquid. Those numbers set up the operational field of the printhead, see Fig. 1.^49,50^ More information is available in the ESI.†1
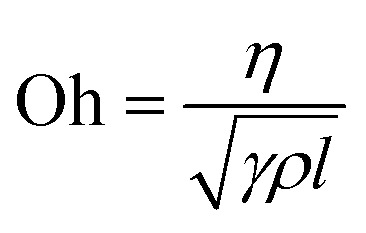
2
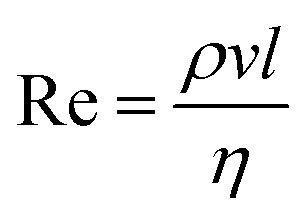


The parameters, which have the main impact on the Oh are viscosity *η* [Ns m^−2^] and surface tension *γ* [N m^−1^] of the liquid. The density *ρ* [kg m^−3^] is roughly the same for all liquids, see Table 1, and the length *l* [m] is the nozzle width (which is also the same for all liquids).

**Table 1:** Testing liquids used in this study and their properties: viscosity (*η*), surface tension (*γ*) and density

Liquid	Water [wt%]	Glycerin [wt%]	Hexanediol [wt%]	Dye [wt%]	Viscosity *η* [mPa s]	Surface tension *γ* [mN m^−1^]	Density *ρ* [kg m^−3^]
High (↑) *η*–low (↓) *γ*	41.9	48	10	0.1	7.2	27.5	1.12
Low (↓) *η*–low (↓) *γ*	64.9	25	10	0.1	3	26.8	1.06
High (↑) *η*–high (↑) *γ*	42.4	57.5	0	0.1	7.99	67.6	1.14
Low (↓) *η*–high (↑) *γ*	74.9	25	0	0.1	1.7	70.5	1.06
Center (⊗) *η*–center (⊗) *γ*	53.4	45	1.5	0.1	4.23	43.5	1.12

To take advantage of the whole operation window of the print head we tried to design liquids that possibly cover the entire printable fluid window in Fig. 1. For example, the upper-left corner has a Oh number of 1 and a Re number of 2, surface tension and viscosity of the testing liquid were chosen to best possibly obtain those values. The final testing liquids were at the very limit of (good) jet-ability, in terms of surface tension and viscosity, indicating that the actual jetting window (dots in Fig. 1) for the employed printhead is somewhat shifted from the region indicated in ref. 49.

### 3.2 Testing liquids

**Fig. 2:**
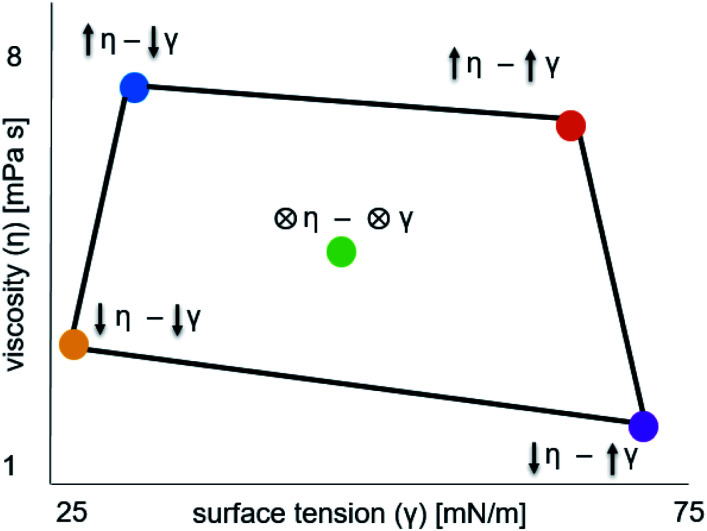
The five testing liquids used in this work, with viscosity (*η*) plotted over surface tension (*γ*). In the labeling of the test points arrows are indicating low (↓) or high (↑) values. Printing of these liquids achieved stable jetting (no severe nozzle blocking, low drop splashing).

In order to find the actual operating window of the printhead the corner points of Fig. 1 were used as starting values. These fluids did not achieve stable jetting and showed the appearance of satellite drops and also severe nozzle blocking. Therefore an adjustment of the liquid composition, was necessary. This fine tuning was done iteratively by jetting new liquids with adapted properties followed by a visual evaluation of the printed pattern in terms of missing dots and dot splashing, done by the operator. Finally five water-based model liquids have been chosen with maximum range in viscosity and surface tension, while still having good jettability and dot quality. The dots in Fig. 1 and 2 show the actual operational window of the print-head.

Viscosity was measured at 25 °C with a Thermo Fisher Haake Rheostress 6000 instrument. The gap width was 0.5 mm, the sample amount was 2 ml and the shear rates were 10, 120, 230, 340, 450, 560, 670, 780, 890 and 1000 Hz.

Surface tension was measured at 25 °C with a Sita science line t60 tensiometer, which uses a bubble pressure method. The bubble lifetime was from 0.05 to 2 seconds. Liquid density was determined using a pyknometer with 25 cm^3^ total volume.

The testing liquids were chosen to have a similar composition like HSI inks. These have water as a main component and also contain larger amounts of higher order alcohols.^51^ We are using glycerine to adjust liquid viscosity and hexanediol to set the surface tension.^52^ 0.1 weight percent of naphthol blue black (98%) were used to colour the model fluids, adding the dye, showed no impact on the liquid properties. It was found out by simple paper chromatography, that the dye travels with the liquid front into the porous media and does not separate from the liquid. Using the dye, drop spreading and penetration can be measured with image analysis. The composition of the liquids is summarized in Table 1.

### 3.3 Papers

There is a wide range of paper grades for inkjet printing available. We investigated four different wood free, fine papers from an industrial supplier, the paper properties are summarized in Table 2. The chosen substrates represent the commercially available papers in the digital printing paper market for office – and HSI papers. The papers' main component is industrial bleached hardwood pulp. The filler used in the papers is scalenohedral PCC (precipitated calcium carbonate).

**Table 2:** Properties of the papers used in this study

Properties	Paper	Paper & primer	Paper & primer & pigments & starch	Hydrophobized paper & starch
HSI surface treatment (primer) [g m^−2^]	0	0	∼0.25	∼0.25
Pigmentation [g m^−2^]	0	0	4	0
Surface starch [g m^−2^]	0	0	∼1	∼1
Hydrophobisation (AKD sizing) [kg t^−1^]	0	0	0	1
Filler content [%]	22	22	23	14
Grammage [g m^−2^]	97.2	78.5	79.89	77.2
Porosity [%]	40.3	38.8	23.6	20.6
Average pore diameter [μm]	3.9	2.6	3.2	4.9
Roughness [ml s^−1^]	321	50	78	191.5
Dispersive surface energy [mJ m^−2^]	44	42	43	33
Polar surface energy [mJ m^−2^]	27	23	22	0.1
Total surface energy [mJ m^−2^]	71	65	65	33.1

• Paper. A plain paper only consisting of pulp and filler. It has no treatment and can be considered as the raw paper, which is the base material for the following three commercial paper grades.

• Paper & primer. This paper has a layer of bivalent salt (=primer) on the surface. This causes faster precipitation of the ink pigments on the paper surface and minimises the travelling of the pigments into the paper bulk.

• Paper & primer & pigments & starch. This coated paper has a low surface pigmentation (4 g per m^2^ per side) of starch and clay. It also has a primer on the surface for better ink setting and a starch layer.

• Hydrophobised paper & starch. The hydrophobised paper has a very low surface energy due to the AKD sizing (see Table 2). On the surface, starch was applied. In this article the words sized and hydrophobised are used as synonyms.

Grammage was measured according to EN ISO 536 and filler content using DIN 54370. The mean pore diameter and the porosity were evaluated with a mercury intrusion porosimetry, a common technique to characterize microscale pore size distributions.^53–55^ We used an Autopore IV 9500 instrument from Micromeritics Instrument Corp.^56^ The roughness was measured according to Bendtsen method ISO 5636-3:2013. The surface energy was measured with a Kruess contact angle device applying the OWRK equation using diiodomethane and water.^57,58^

### 3.4 Print through test (PT)

**Fig. 3:**
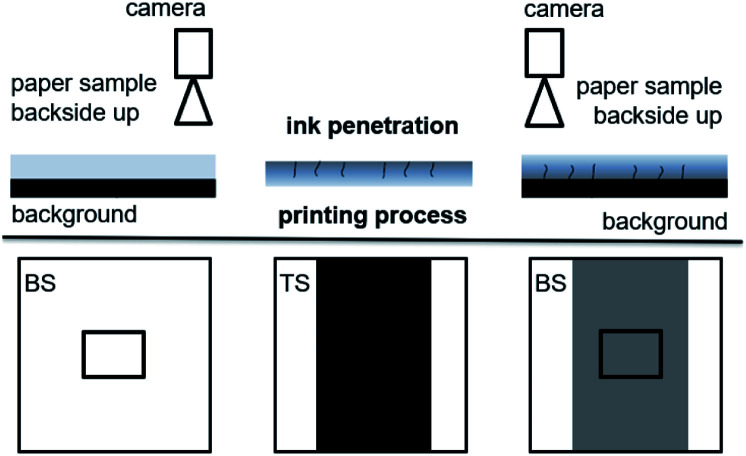
Measurement principle of the print through test. An image of a 5 × 5 cm^2^ area is taken of the backside (BS) of the paper. Then the print is applied on the topside (TS) of the paper. After printing, the exactly same 5 × 5 cm^2^ area of the BS of the now printed sheet is imaged. The difference in gray value before and after printing is the print through.

The print through method is used to evaluate the print ink penetration into the paper. It measures the colour difference before and after printing on the backside of the paper, see Fig. 3. Before and after printing same area of the paper is investigated. The higher the difference, the higher the ink penetration into the paper. A more detailed description of this method can be found in the ESI. GV denotes the mean gray value [8 bit grayvalue] of the measurement area.3
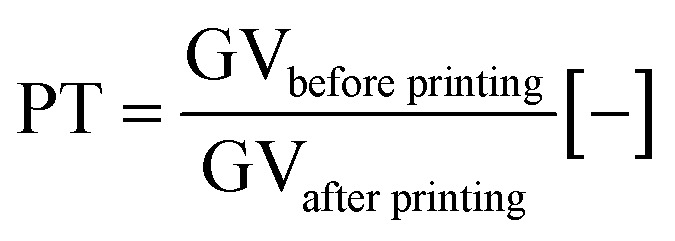


### 3.5 Image analysis of printed dots

The ImageXpert set up uses an overhead camera with a manual stage for repeatable sample positioning.

**Fig. 4:**
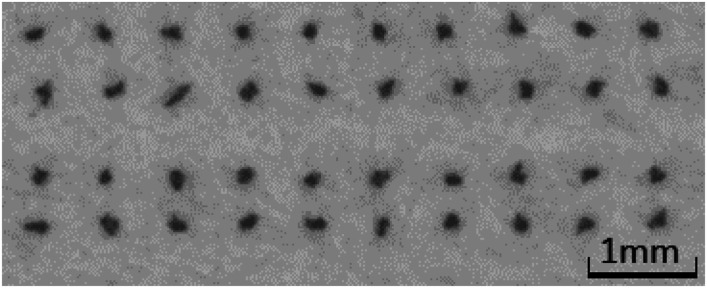
Picture of the printed dot pattern. The variability of the drop area is generated by the heterogeneity of the paper.

Image analysis of the printed dots was carried out using the ImageXpert image analysis system. It records images of the print and provides fully automated digital image analysis of the printed dots. A picture of such a dot pattern can be seen in Fig. 4. For further information on image analysis and experimental error please read the ESI.† We used the parameters dot area (*A*), gray average (GA) and axis ratio (AR). Gray average GA is the mean gray value [8 bit GV] of the dot. The darker the drop, the lower is the gray average. The area of the droplet is a parameter for drop spreading. The second parameter analysed from the prints is the light absorption LA of the individual printing dots.

4 LA = ΔGA × *A* [8 bit grayvalue mm^2^]

We calculate the light absorption using the difference ΔGA between the (higher) gray average of the paper and the (lower) gray value of the dot, the unit of ΔGA is [8 bit grayvalue]. ΔGA is multiplied with the measured drop area *A*, which defines the unit of LA as [8 bit grayvalue mm^2^]. Digital cameras are linear sensors, when the gamma value *γ* of the camera is set to *γ* = 1. Hence ΔGA is linear proportional to the light remission of the dot, and LA = ΔGA × *A* is the amount of light absorbed by the printed dot.

## 4 Results

### 4.1 Drop spreading and dot area

**Fig. 5:**
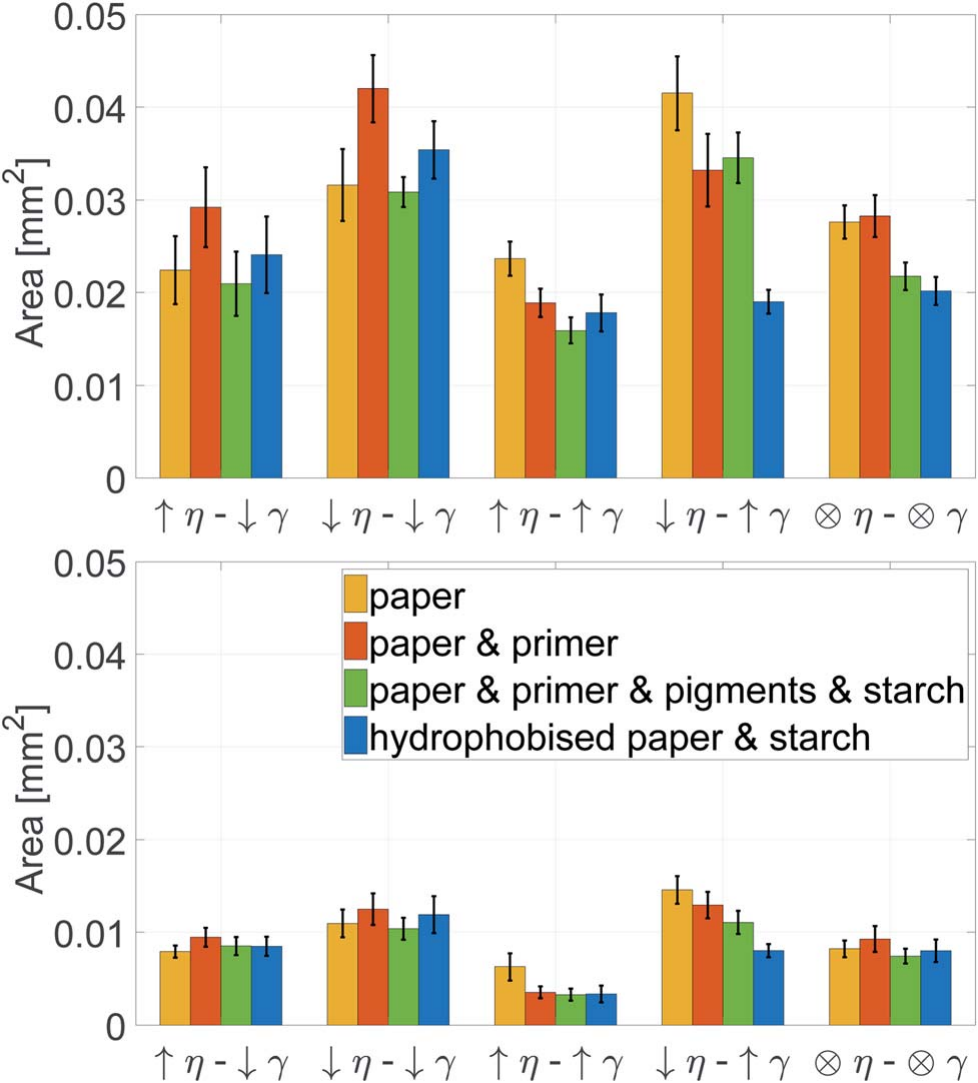
Inkjet printed dot area for 120 pl (top) and 30 pl (bottom) drop size. A large dot area indicates more intense spreading of the liquid drop. The printing liquid has high (↑), medium (⊗) or low (↓) viscosity (*η*) and surface tension (*γ*) as described in Table 1. For details on the printing substrates see Table 2. Bars are 95% confidence limits. Low viscosity increases the spreading. Surface tension plays a minor role as long as the substrate is not hydrophobised. Here, low surface tension eases spreading.

Fig. 5 shows the dot area for two drop sizes, 120 pl (top) and 30 pl (bottom). A larger dot area indicates more drop spreading. Generally, it can be observed that the results of 120 pl and 30 pl are very similar, indicating good repeatability and statistical significance of the measurements. The liquid, which exhibits low surface tension *γ* and low viscosity *η* shows the highest spreading. However, the fluid with high *γ* and low *η* also reveals high dot area, indicating that a low liquid viscosity is the relevant factor for drop spreading. Surface tension seems to play a much smaller role, except for the hydrophobised paper. The effect of viscosity and surface tension is much higher for the 30 pl drops, where the difference in drop size is 300% between the lowest and highest values, whereas for the 120 pl drops the maximum difference is 200%.

**Fig. 6:**
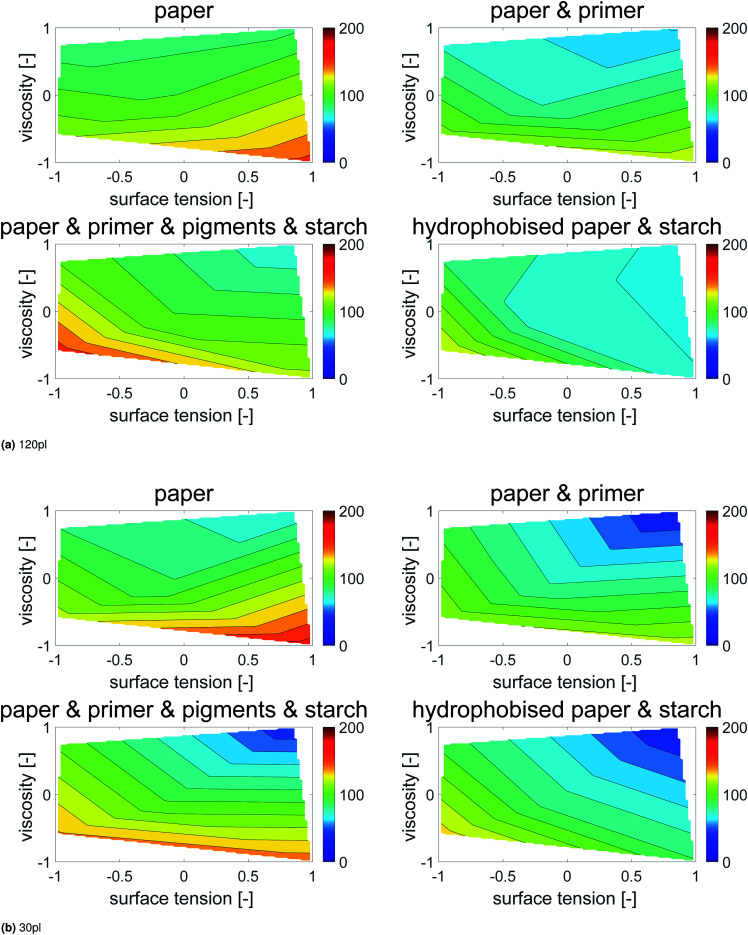
Contour plots of printed dot area (in % of mean area per dot size) over dimensionless surface tension and viscosity. Each plot represents one of the papers from Table 2. The contour lines represent combinations of *γ* and *η* resulting in equivalent dot area. The almost horizontal lines illustrate a predominant impact of viscosity on the dot area. The surface tension effect plays a minor role except for the sized sheet (bottom, right), indicated by the changing slopes of the contour lines into a more vertical direction.

Contour plots were generated, where the dot area is plotted over surface tension (*x*-axis) and viscosity (*y*-axis), Fig. 6. Values between the 5 measurement points were interpolated linearly. All points with the same dot area are connected to a contour line, which represents the same dot area for different combinations of surface tension and viscosity. The impact of viscosity and surface tension on the dot area can be seen by the partial derivative (*i.e.* the slope) of the surface in *x*- and *y*-direction. A high slope in *x*-direction indicates a high influence of surface tension, a high slope in *y*-direction indicates a high influence of viscosity. Also in this plot the main effect on dot area is liquid viscosity, represented by the almost horizontal contour lines. In Fig. 6 one can better see that for the hydrophobised sheets, surface tensions has a stronger influence, as indicated by the change of contour line slopes which are more tilted towards the *x*-axis.

In order to quantify the impact of surface tension and viscosity, we calculated the mean partial derivative, with respect to viscosity, eqn (5), and surface tension, eqn (6), for each point in the contour plots.5
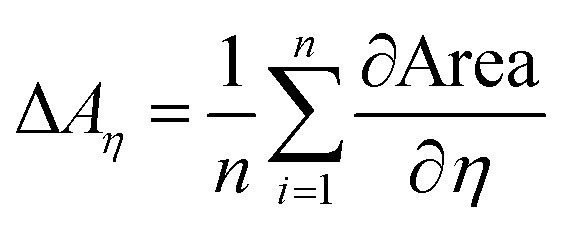
6
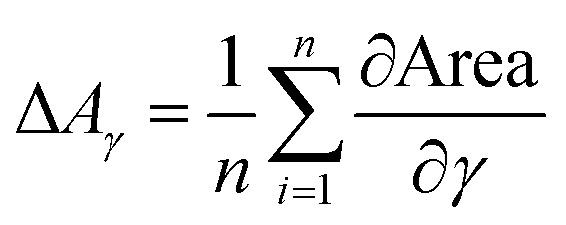


**Fig. 7:**
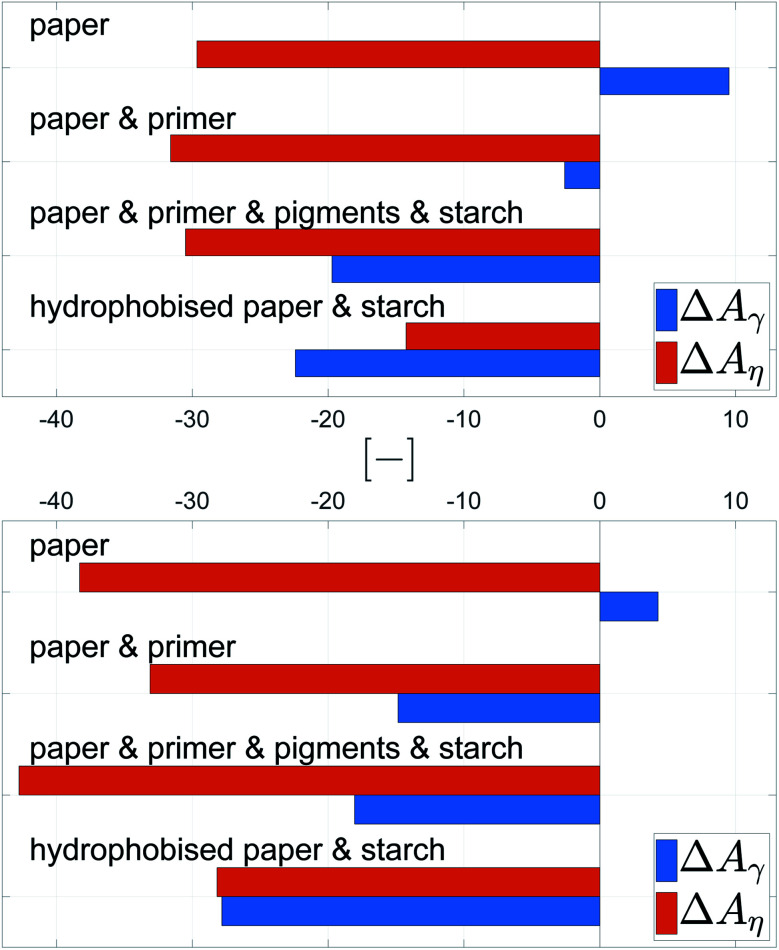
Average effect of viscosity (Δ*A*_*η*_, orange bar) and surface tension (Δ*A*_*γ*_, blue bar) on printed dot area *A* for 120 pl drops (top) and 30 pl drops (bottom). Positive values indicate an increase of the printed dot area for higher surface tension *γ* and viscosity *η* (negative values indicate a decrease), compare eqn (5) and (6).

The mean of the partial derivatives is plotted in Fig. 7. As expected higher viscosity leads to lower dot area, indicated by the negative values. Surface tension reduces dot area, except for the plain paper, where it has a very small opposite effect. The change in viscosity has a stronger impact on the final dot area, than surface tension. This holds for both droplet sizes, except for the hydrophobised paper. Here, the surface tension plays a more important role than viscosity.

The values for surface tension and viscosity in Fig. 6 are mapped to a range from −1 to 1, representing the respective minimum/maximum values in the jetting window as shown in Fig. 2 and Table 1. The mean partial derivatives Δ*A*_*η*_ and Δ*A*_*γ*_ in eqn (5), (6) and Fig. 7 thus give the impact of surface tension and viscosity on dot area in terms of the variation possible within the jetting window. That means that *e.g.* a value of −30 for ∂*A*/∂*η* indicates a twice as high influence when changing viscosity from the center point to the maximum value than *e.g.* a value of −15 for ∂*A*/∂*γ* giving the influence of changing surface tension from the center point to the maximum value.

Three main findings for high speed inkjet printing can be derived from these results. First, viscosity has the main impact on final dot area of the printed drops, high viscosity causes low dot area. Second, surface tension plays a minor role except for liquids which are used for jetting on a hydrophobized substrate (sized paper). This reveals, that special focus needs to be put on surface tension, when printing on hydrophobised materials. Finally, the change of liquid property has an affect of up to a factor of 200% between the area of low and high surface tension and viscosity values. So by adjusting the liquid properties, a 2 times higher printed dot area can be achieved for 120 pl droplets. This effect gets even more pronounced for smaller droplets, for the 30 pl drops there is a factor of 300%.

### 4.2 Liquid penetration

**Fig. 8:**
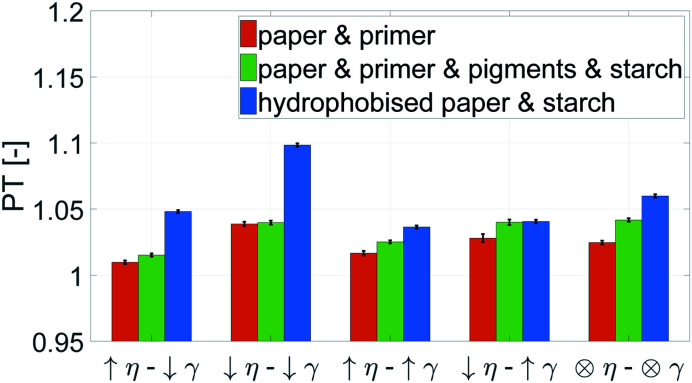
Print through index PT of papers with the same grammage. A high value represents a strong penetration of the liquid into the paper. The liquid properties can be found in Table 1. Low viscosity *η* increases the penetration. For the hydrophobised samples also low surface tension *γ* favours strong imbibition into the paper.

The stronger the penetration into the paper, the higher is the print through (PT) value, as described in Section 3.4. Fig. 8 shows, that the liquids with low surface tension and low viscosity show the highest penetration. Especially for the hydrophobised paper, low surface tension causes a deep penetration of the liquid. The liquids with high viscosity have the smallest print through for all three substrates, indicating that low viscosity aggravates liquid penetration.

**Fig. 9:**
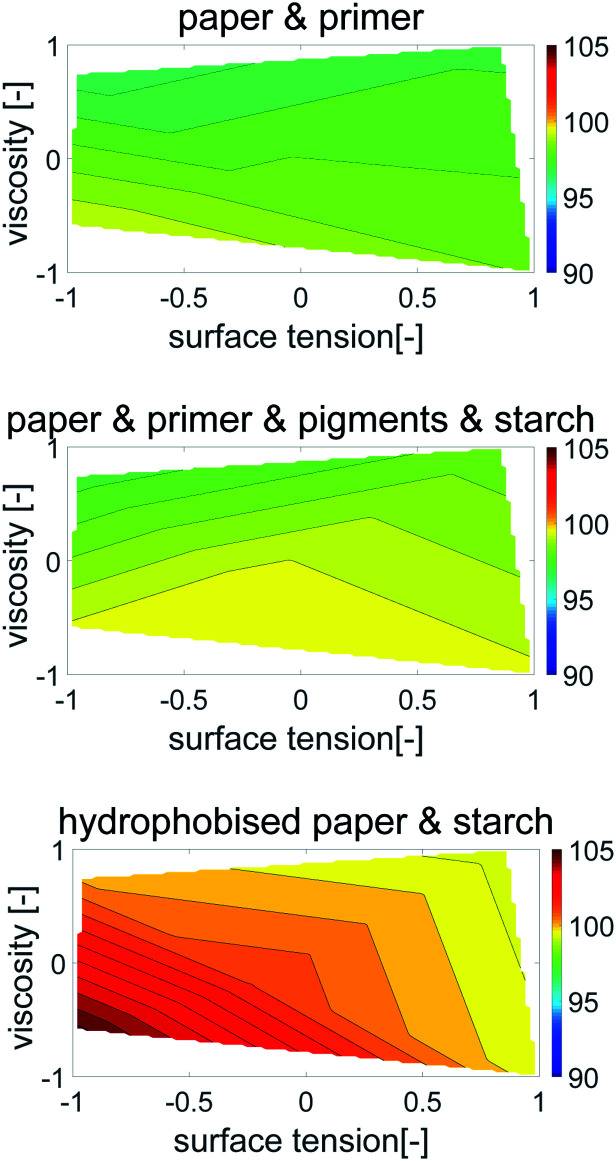
Contour plots of print through value over surface tension *γ* and viscosity *η*. Each plot illustrates one substrate from Table 2. The contour lines represent combinations of *γ* and *η* resulting in same print through. The horizontal lines show a strong influence of viscosity for the unsized papers. The sized sheets are influenced by both parameters, viscosity and surface tension (bottom), indicated by the changing slopes of the contour lines into a less horizontal direction.

Again a contour plot was generated, now with liquid penetration over viscosity on the *y*-axis and surface tension on the *x*-axis, see Fig. 9. In a similar manner, like above, the mean partial derivative of print through ΔPT_*η*_ with respect to viscosity (eqn (7)) and ΔPT_*γ*_ with respect to surface tension (eqn (8)) was calculated from the contour plots7
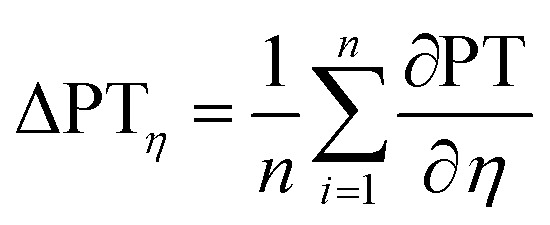
8
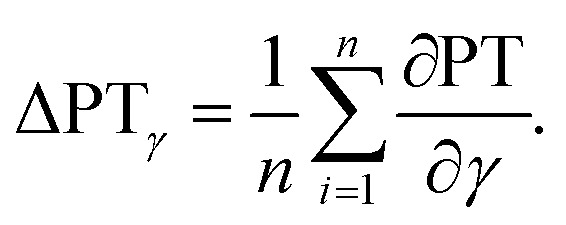


**Fig. 10:**
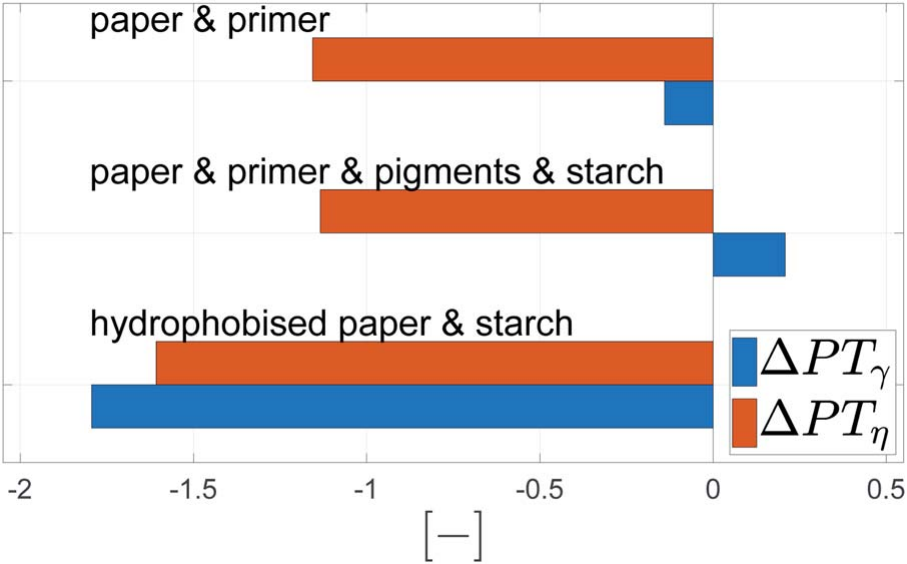
Mean impact of viscosity (ΔPT_*η*_, orange bar) and surface tension (ΔPT_*γ*_, blue bar) on penetration into the paper. The bigger the bar, the stronger the influence of the represented property. Negative values show a decrease of the print through value for higher surface tension *γ* and viscosity *η* (positive values show an increase), compare eqn (8) and (7).

The mean partial derivatives are plotted in Fig. 10, quantifying the influence of viscosity and surface tension change on the print through. The viscosity has a strong influence on penetration for all papers as indicated by the high slope in *y*-direction in the contour plots, Fig. 9. Low viscosity leads to high ink penetration, which is plausible. The impact of surface tension is inconsistent for the unsized papers, the contour lines are pointing upwards and downwards in these graphs, indicating a contradictive effect on liquid penetration. Consequently the mean partial derivative ∂PT/∂*γ* is low, as no systematic effect of surface tension can be found. The hydrophobised substrate shows a high impact from both parameters. Lower surface tension and lower viscosity cause an increase in penetration and thus a higher PT value.

In summary, for all substrates liquid penetration is strongly promoted by low viscosity. For hydrophobised papers, surface tension is equally important, low surface tension promotes liquid penetration.

### 4.3 Surface coverage

In order to evaluate surface coverage we measure light absorption (LA). LA of individual printing dots is calculated as the product of the area of the printed dot and the difference of the gray average (GA) between paper and printed dot, eqn (4). A high LA value corresponds to a darker colour and/or higher area of the dot. In this way LA provides information on the degree of liquid surface coverage, *i.e.* good liquid spreading with little liquid penetration.

**Fig. 11:**
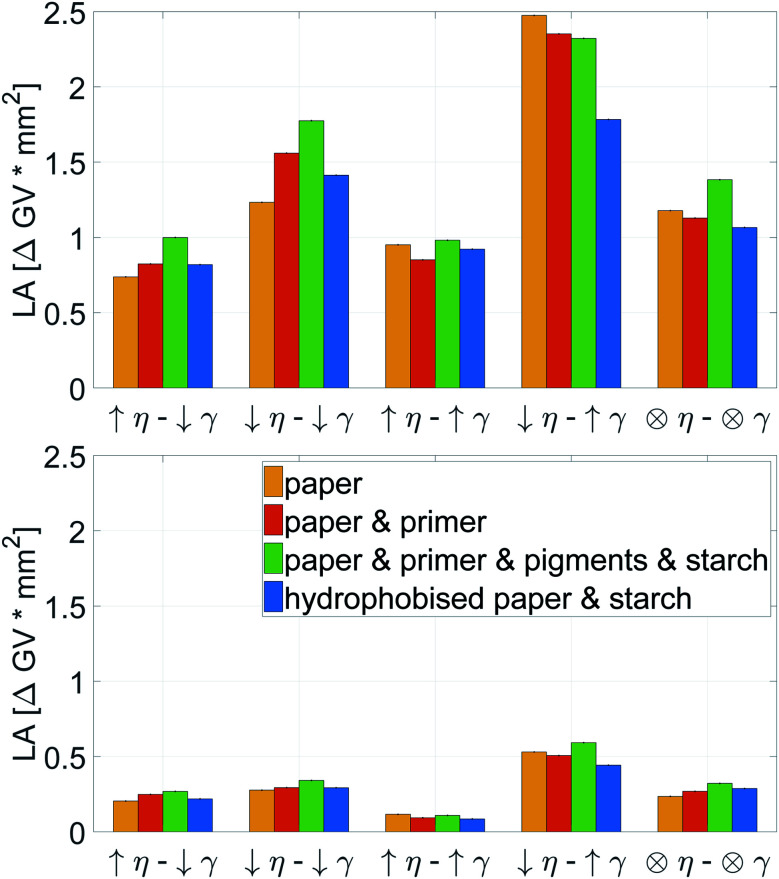
Light absorption for 120 pl (top) and 30 pl (bottom) drop size. The printing liquid has high (↑) or low (↓) viscosity (*η*) and surface tension (*γ*) as described in Table 1. The ⊗ sign represents viscosity and surface tension value from the center point. For details on the printing substrates see Table 2. Bars are 95% confidence limits. The viscosity has a major impact on all substrates and droplet sizes. Low viscosity favors high LA.

The highest light absorption is obtained by liquids with low viscosity and high surface tension, see Fig. 11. This is valid for big and small droplet sizes. For the small drops high viscosity and high surface tension causes an extremely low light absorption. For light absorption, the substrate influence is of lower importance, the values are fairly similar for all investigated papers.

**Fig. 12:**
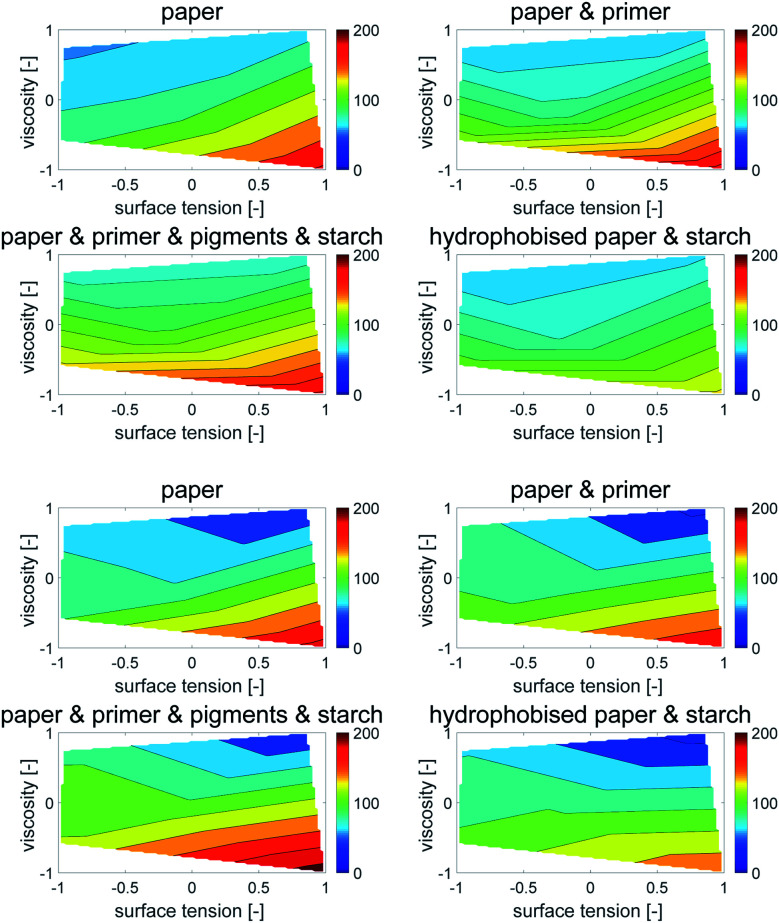
Contour plots of light absorption over surface tension *γ* and viscosity *η*. (120 pl dots top, 30 pl dots bottom) Each plot illustrates one of the papers from Table 2. The contour lines represent combinations of *γ* and *η* resulting in equivalent light absorption. The fairly horizontal lines show a strong impact of viscosity on the light absorption.

Also here contour plots were made, see Fig. 12. For all different paper treatments and the drop sizes the shape of the contour plots is similar, the lines are fairly horizontal, indicating the predominant influence of viscosity on dot light absorption. Again the mean partial derivatives with respect to viscosity (eqn (9)) and surface tension (eqn (10)) of the data points in the contour plots were computed.9
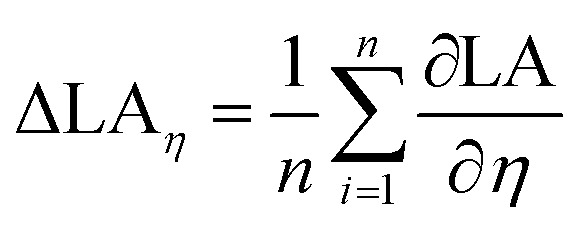
10
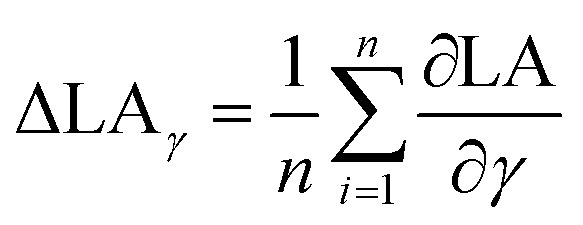


**Fig. 13:**
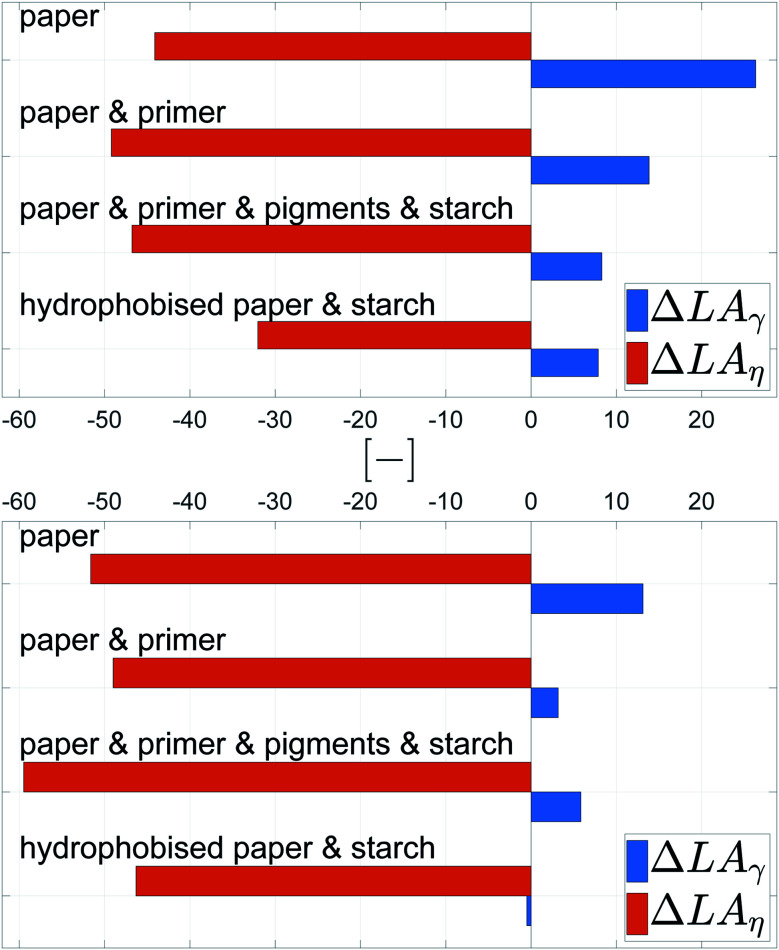
Average influence of viscosity ΔLA_*η*_ and surface tension ΔLA_*γ*_ on the light absorption for 120 pl (top) and 30 pl (bottom) drops. Light absorption is a measure for good surface coverage. The positive values for ΔLA_*γ*_ indicate a moderate increase of dot light absorption for higher surface tension *γ*, especially for the larger drops. The negative values for ΔLA_*η*_ demonstrate a considerable negative impact of viscosity on dot light absorption.

Fig. 13 gives the mean values ΔLA_*η*_ and ΔLA_*γ*_, quantifying the impact of viscosity and surface tension on dot light absorption. All substrates show the predominant influence of the viscosity, Fig. 13, ΔLA_*η*_ is at least by the factor 3 higher than ΔLA_*γ*_. However, the surface tension still has an impact on the light absorption, especially for the bigger droplet size. A decrease of viscosity causes an increase of light absorption, whereas an increase in surface tension results in a light absorption increase. This observations hold for all four substrates, irrespective of the surface treatment of the paper.

For increasing color density in low and medium printing tone values in HSI printing reduction of liquid viscosity (and to a lower extent increase of liquid surface tension) is a promising strategy. In order to develop the full tone density, however our results suggest to increase viscosity, and for sized papers also increase surface tension, as this will reduce liquid penetration. In terms of print gamut, our results suggest that liquid viscosity governs a tradeoff between higher gamut in the light and middle tones (low viscosity) and higher gamut in the full tones (high viscosity).

In summary, the liquid surface coverage (light absorption) is mainly influenced by viscosity, to some extent surface tension is also relevant, especially for larger drops. Higher LA is achieved by reducing viscosity and increasing surface tension. The substrate plays a minor role, especially for small droplets. It is remarkable that, irrespective of surface treatment (hydrophobisation – paper sizing, pigmentation and inkjet surface treatment with salt) the effect of viscosity on dot light absorption is stable and nearly equally strong.

## 5 Conclusions

In the current work we have investigated the influence of liquid- and substrate properties on high speed inkjet printing outcome. We have chosen representative process conditions within the inkjet operating window in terms of picoliter drop size, liquid surface tension and viscosity.

Three main characteristics of the resulting inkjet printing have been analyzed: drop spreading on the surface (dot area), liquid penetration (print through) and the surface coverage of the individual printing dots, measured by light absorption.

### 5.1 Drop spreading

Altogether, the measured dot area showed that for both picoliter droplet sizes, the viscosity is the main impact factor. Lower viscosity results in higher spreading and thus a larger dot area. Only for the hydrophobised substrate (sized paper), the surface tension has a considerable influence. Here, the decrease of surface tension causes an increase in spreading.

### 5.2 Liquid penetration

The print through shows a strong dependence on viscosity, except for the hydrophobised sample which, again, is heavily influenced by surface tension. Similar to dot spreading, high viscosity causes low penetration of the liquid.

### 5.3 Surface coverage

Surface coverage is the combination of good liquid spreading with little liquid penetration into the substrate, we measured it with light absorption. Again viscosity turned out to be most important, it reduces the surface coverage. However, also a decrease in surface tension was found to cause a decrease in surface coverage. These findings are equivalent for all four substrates and for all drop sizes, however the influence of surface tension is less for smaller drops.

The surface coverage (light absorption) is a result of two competing processes, dot spreading and liquid penetration. As shown above both of these factors are promoted by the same liquid properties, mostly low viscosity but also low surface tension. Considering that low viscosity is also the driving factor for surface coverage of the printed dot it becomes clear, that the increase in dot spreading is clearly dominating over the increase in liquid penetration for the investigated dot sizes. That means that in order to obtain a good surface coverage in an inkjet printing process on porous substrates, reducing the viscosity and increasing the surface tension is a promising strategy. These findings can also be transferred to other inkjet applications on porous surfaces like printed electronics,^9^ patterning of microfluidic sensors,^59,60^ or membrane functionalisation.^61^

### 5.4 Surface tension and viscosity

We would like to point out, that our results provide a quantitative analysis of viscosity and surface tension impact on HSI print. The most important general finding here is that for liquids within the jetting window the variation of the liquid viscosity typically has a 2–3 times higher impact on the print outcome than variation of the liquid surface tension. Viscosity in all cases reduces dot area, liquid penetration and dot surface coverage. Surface tension significantly reduces dot spreading and liquid penetration of hydrophobised substrates, however it consistently increases dot surface coverage for all papers and drop sizes.

## Conflicts of interest

There are no conflicts to declare.

## Supplementary Material

RA-009-C9RA04993B-s001
