# Supplementary material for: The effect of viscosity and surface tension on inkjet printed picoliter dots
Source: RSC Adv. 2019 Oct 7;9(54):31708–19. doi: 10.1039/c9ra04993b (PMC9072721; doi:10.1039/c9ra04993b)
Supplement: RA-009-C9RA04993B-s001 [file RA-009-C9RA04993B-s001.pdf]

Electronic Supplementary Material (ESI) for RSC Advances.
This journal is © The Royal Society of Chemistry 2019

Electronic Supplementary Information to "The Effect of Viscosity and Surface Tension on High Speed Inkjet Printed Picoliter Dots"

1 Materials and Methods

1.1 HSI printhead and drop volume

Evoked from the different viscosity and surface tension of the liquids, the produced droplets have some deviation in their volume when using the same print head settings as frequency and electric wave form. Therefore it was necessary to measure the exact drop volume at each print setting. 500 sheets were printed with a defined dot pattern. The difference in reservoir liquid before and after printing divided by the amount of droplets was used to calculate the actual average drop volume for each setting. The deviation from the target drop size was at maximum 5% for the 30pl drops and 3.3% for the 120pl drops.

1.1.1 Operational Field of the HSI printhead

The Oh number helps to predict fluid flow break up and thus droplet formation. It is the ratio between viscous forces to surface tension and inertia. A higher value indicates a danger for nozzle blocking due to e.g. a too viscous liquid (Figure 1 in the main article, blue region), where as a too low Oh might cause drop splashing and satellite drops (Figure 1 in the main article, red region and area between dashed line and right corner)). According to literature, fluids with an Ohnesorge value Oh between 0.1 and 1 are jetable in HSI printing. The Reynolds (Re) number relates the inertial to the viscous forces. The characteristic length l [m] is the nozzle width (0.02 mm). The speed of the droplets was constant at circa $8m/s$. Above the critical Re number the laminar flow turns into a turbulent flow, the critical value for a tube flow is around 2300. In the HSI field, the Re number is stated to be between 2 and 160 to have a printable fluid (see Figure 1 in the main article) [1]. A too low Reynolds number can result in a continuous liquid jet without separation of the drops, see Figure 1 in the main article, green region, in the main article.

1.2 Print through test (PT)

First a quadratic 5x5cm^2 square on the backside (BS in Figure 3 in the main article) of the unprinted paper is imaged in front of a coloured background. It is important that the colour of this background is identical to the colour of the test ink. This image gives the pure shine through of the coloured background without any ink penetration. Then the paper is printed on the top side (TS

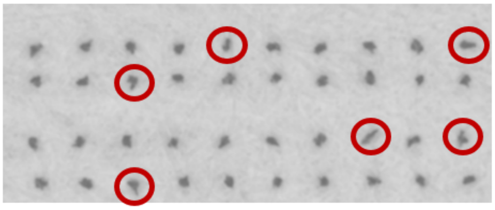

Figure 1: Picture of the printed dot pattern. The dots with an axis ratio below 0.85 are excluded and marked with a red circle. The variability of the drop area is generated by the heterogeneity of the paper.

in Figure 3 in the main article) and the same $5x5cm^2$ area of the backside of the now printed paper is imaged again (shown on the right side of Figure 3 in the main article). The images are taken using a microscope (Alicona Infinite Forcus) with diffusive illumination to eliminate surface shading effects due to the paper roughness. The final image of size $5x5cm^2$ is stitched together from 10x10 overlapping images taken by the instrument. Illumination artifacts were removed by frequency domain image filtering.

The ink penetration index PT [-] is calculated from the ratio between the gray value of the unprinted sample backside image to the gray value of the printed sample backside image, Equation 3 in the main article.

1.3 Image analysis of printed dots

The used papers have a gray average of around 220. Axis ratio AR is a shape descriptor of the dot, where two perpendicular axes are fit to the dot area and AR [-] is the ratio of the shorter axis length to the longer axis length. A value of 1 describes a perfectly round object, increasingly lower values indicate a more and more elongated shape.

A printed drop pattern is shown in Fig. 1. Despite detailed evaluation of the printed dot pattern for developing liquids with good jetability, there is still some drop splashing. Another point, which needs to be considered is, that due to small droplet size and low color density of the dots, the dot area on the paper surface is not always detected correctly. Thus we decided to only evaluate dots with an aspect ratio higher than 0.85. This filter eliminates all dots which are fragmented due to image segmentation problems or exhibit drop splashing (i.e. dots with satellite drops), which are diluting the measurement results. Figure 1 shows the dots as printed in the trial. The picture also indicates drops with AR below 0.85 (red circles), which are not included in the evaluation. The area of the droplet is a parameter for drop spreading.

Please note that liquid penetration and liquid spreading have the opposite effect on light absorption, LA increases with drop spreading (higher dot area A) and decreases with liquid penetration (lighter dot, hence lower ΔGA).

1.3.1 Experimental Error

Local variation of paper properties within one sheet is large, leading to large variations in the local print appearance [2], which has e.g. been shown for local print density [3] and local printing ink penetration [4]. Thus for quantitative evaluation of print results it is mandatory to test a large enough specimen area or several smaller areas sampled from different regions of the paper. We printed each liquid on 6 different sheets from the same paper grade and analysed 3 to 7 regions of this printed area with at least 1.5 centimeters space between these regions. Excluding droplets with an aspect ratio less than 0.85, we ended up with an average amount of 200-600 droplets per liquid paper combination. The difference is caused by the droplet size itself, bigger droplets need more area than the smaller ones and thus less drops can be printed in one region with one nozzle firing. We computed 95% confidence limits for each test result by counting each evaluated region as one specimen, the number of independent samples per test point therefore was between 18 and 42, considering that we tested 6 sheets with 3-7 regions each.

References

[1] G. H. McKinley and M. Renardy. Wolfgang von Ohnesorge. *Physics of Fluids*, 23(127101), 2011.

[2] U. Hirn, M. Lechthaler, and W. Bauer. Registration and point wise correlation of local paper properties. *Nordic Pulp and Paper Research Journal*, 23(4):374–381, 2008.

[3] W. Fuchs, M. Dauer, U. Hirn, and W. Bauer. A memory effect in sheet-fed offset printing. In *Paper Conference and Trade Show, PaperCon 2017: Renew, Rethink, Redefine the Future*, volume 2, page 1098, 2017.

[4] C. Kappel, U. Hirn, M. Donoser, and W. Bauer. Measurement of Printing Ink Penetration in Uncoated Papers and Its Influence on Print Quality. *94th Annual Meeting, Pulp and Paper Technical Association of Canada*, pages 539–542.
